# Variations in Gut Microbiome Are Associated with Prognosis of Hypertriglyceridemia-Associated Acute Pancreatitis

**DOI:** 10.3390/biom11050695

**Published:** 2021-05-06

**Authors:** Xiaomin Hu, Liang Gong, Ruilin Zhou, Ziying Han, Li Ji, Yan Zhang, Shuyang Zhang, Dong Wu

**Affiliations:** 1 State Key Laboratory of Complex Severe and Rare Diseases, Department of Medical Research Center, Peking Union Medical College Hospital, Chinese Academy of Medical Sciences & Peking Union Medical College, Beijing 100730, China; huxiaomin2015@163.com; 2 State Key Laboratory of Complex Severe and Rare Diseases, Department of Gastroenterology, Peking Union Medical College Hospital, Chinese Academy of Medical Sciences & Peking Union Medical College, Beijing 100730, China; gongl_pumc@163.com (L.G.); han-zy16@mails.tsinghua.edu.cn (Z.H.); jili@student.pumc.edu.cn (L.J.); 3 Department of Cardiology, Peking Union Medical College Hospital, Chinese Academy of Medical Science & Peking Union Medical College, Beijing 100730, China; zrl14@mails.tinghua.edu.cn (R.Z.); shuyangzhang103@nrdrs.org (S.Z.); 4 Institute of Cardiovascular Sciences and Key Laboratory of Molecular Cardiovascular Sciences, School of Basic Medical Sciences, Ministry of Education, Peking University Health Science Center, Beijing 100191, China; zhangyan9876@pku.edu.cn

**Keywords:** acute pancreatitis, gut microbiota, hypertriglyceridemia, prognosis

## Abstract

Hypertriglyceridemia-associated acute pancreatitis (HTGAP) is linked with increased severity and morbidity. Intestinal flora plays an important role in the progression of acute pancreatitis (AP). However, pathogenetic association between gut microbiota and HTGAP remains unknown. In this study, we enrolled 30 HTGAP patients and 30 patients with AP that is evoked by other causes. The V3–V4 regions of 16S rRNA sequences of the gut microbiota were analyzed. Clinical characteristics, microbial diversity, taxonomic profile, microbiome composition, microbiological phenotype, and functional pathways were compared between the two groups. Our results showed that the HTGAP group had a higher proportion of severe AP (46.7% vs. 20.0%), organ failure (56.7% vs. 30.0%), and a longer hospital stay (18.0 days vs. 6.5 days). HTGAP group also had poorer microbial diversity, higher abundances of *Escherichia/Shigella* and *Enterococcus*, but lower abundances of *Dorea longicatena*, *Blautia wexlerae*, and *Bacteroides ovatus* as compared with non-HTGAP group. Correlation analysis revealed that gut bacterial taxonomic and functional changes were linked with local and systemic complications, ICU admission, and mortality. This study revealed that alterations of gut microbiota were associated with disease severity and poor prognosis in HTGAP patients, indicating a potential pathophysiological link between gut microbiota and hypertriglyceridemia related acute pancreatitis.

## 1. Introduction

Acute pancreatitis (AP) is one of the most common gastrointestinal diseases requiring acute hospitalization, with an annual incidence of 34 per 100,000 [1,2]. AP is characterized by local and systemic inflammatory responses, and it is graded into mild AP (MAP), moderately severe AP (MSAP), and severe AP (SAP) according to the 2012 revised Atlanta Classification [3]. AP’s overall mortality was 1~3%, but it reached up to 30% in patients with SAP [4].

Gallstone (45%) remains the leading cause of AP, followed by alcohol abuse (20%) [1]. Speck first reported the etiological link between hypertriglyceridemia and AP in 1865 [5]. However, with rapid economic growth and a shift in diet structure, the incidence of hypertriglyceridemia-associated acute pancreatitis (HTGAP) keeps increasing worldwide [6,7]. The pathophysiological mechanism of HTGAP remains largely unknown, but animal studies suggest that the accumulation of free fatty acids and overactivation of the inflammatory response may be responsible for the onset of this disease [8]. An increasing body of evidence indicates that HTGAP has a higher severity than non-HTGAP, including higher risks of infected necrosis, organ failure, ICU admission, longer hospitalization, and death [9,10,11]. The current management for HTGAP includes supportive therapy of organ dysfunction, fluid resuscitation, pain control, nutritional support, insulin infusion, anti-hypertriglyceridemia medications, heparin, hemofiltration, and plasmapheresis [8]. However, no standard management modality or guideline for HTGAP is available.

In recent years, studies have indicated that the changes of intestinal microflora were closely related to AP’s progression. For instance, AP patients had a higher relative abundance of potentially pathogenic bacteria, such as *Enterobacteriaceae* and *Enterococcus*, and a lower relative abundance of beneficial bacteria, such as *Bifidobacterium* as compared with healthy controls [12]. Multiple factors lead to AP’s progression and aggravation, including the imbalance of bacterial composition, decreased production of short-chain fatty acids (SCFAs), disruption of intestinal mucosal barrier function, and translocation of intestinal bacteria [13]. Our previous studies revealed different gut microbiota compositions and functions in AP patients with different severity [14]. Yun et al. reported that hypertriglyceridemia was associated with decreased gut microbiota diversity, which might be an important target for regulating blood lipids [15]. A recent systematic review showed that SAP patients treated with pre/pro/synbiotics had a lower risk of organ failure and a shorter duration of hospital stay [16]. However, the link between intestinal microbiota and the prognosis of HTGAP remains unknown. Therefore, investigating the pathogenetic role of altered gut microbiota in HTGAP patients enriches our knowledge about AP and provides opportunities to improve current therapy.

This study aims to investigate the relationship between the changes of intestinal flora and the prognosis of HTGAP patients, and to lay a basis for future research and translation.

## 2. Materials and Methods

### 2.1. Study Population

This was a single-center, prospective, observational cohort study. Between June 2018 and April 2019, we enrolled AP patients in Peking Union Medical College Hospital, Beijing, China. A stratified random sampling method was utilized to select the patients from our database for this study. The inclusion criteria included a diagnosis of AP according to the 2012 revised Atlanta criteria [3]. Patients must be present in the hospital within 24h after disease onset. The major exclusion criteria were chronic pancreatitis, immunosuppressive disease, inflammatory bowel disease, cancer, irritable bowel syndrome, gastroenteritis, narcotizing enterocolitis, and use of antibiotics, probiotics, laxatives, or Chinese herbs within two months of enrollment. Informed consent was obtained from all of the participants. Ethical approval was gained from the Ethics Committee of PUMCH (Identifier: JS1826, Date of approval: 20th February 2018. Period of validity: February 2018 to August 2020).

### 2.2. Demographic and Clinical Data

We reviewed patients’ electronic medical records to obtain demographic and clinical data, which included age, gender, height, weight, combined diseases, laboratory data, imaging examinations, disease severity, complications, and clinical outcomes. HTGAP was defined as serum triglycerides >1000 mg/dL and excluding other etiologies of AP [17]. According to the 2012 revised Atlanta Classification [3], AP was graded into MAP, MSAP, and SAP. The Acute Physiology and Chronic Health Evaluation II (APACHE II) [18], the Sequential Organ Failure Assessment (SOFA) scores [19], and Balthazar scores [20] were also obtained to evaluate the severity of the disease. The definitions of local and systematic complications were based on previous studies [3,21]. Local complications included acute peripancreatic fluid collection (APFC), pancreatic pseudocyst, acute necrotic collection (ANC), walled-off necrosis (WON), and infected necrosis. APFC was defined as peripancreatic fluid collections with homogeneous liquefied components and without well-defined walls. Pancreatic pseudocyst was an encapsulated collection of fluid with a well-defined inflammatory wall that is usually outside the pancreas with minimal or no necrosis. ANC was a collection containing variable amounts of both fluid and necrosis associated with necrotizing pancreatitis. WON was a mature and encapsulated collection of pancreatic and/or peripancreatic necrosis that has developed a well-defined inflammatory wall. Infected necrosis was defined as a positive culture of pancreatic or peripancreatic necrosis that was obtained by fine-needle aspiration (FNA) or the presence of gas in the fluid collection on contrast enhanced CT. Systematic complications comprised acute respiratory distress syndrome (ARDS), systemic inflammatory response syndrome (SIRS), acute kidney injury (AKI), abdominal compartment syndrome (ACS), liver damage, myocardial injury, altered mental status, sepsis, shock, and bowel obstruction. ARDS was defined as respiratory dysfunction that is characterized by acute onset, bilateral opacities on chest radiography, and a PaO2/FiO2 ≤ 300 with a final expiratory pressure or continuous positive airway pressure ≥ 5 cmH2O. SIRS was diagnosed if two of the following criteria are met: respiratory rate >20 breaths/min., heart rate >90 beats/min., leucocyte count >12,000 cells/μL or <4000 cells/μL, and body temperature >38 or <36 °C. AKI was diagnosed if two of the following criteria are met: an increase in serum creatinine by ≥0.3 mg/dL (≥26.5 mmol/L) within 48 hours, increase in serum creatinine to ≥1.5 times baseline within the prior seven days, and urine volume of <0.5 mL/kg/hour for six hours. ACS was defined as a sustained intra-abdominal pressure >20 mmHg (with or without abdominal perfusion pressure <60 mmHg) that is associated with new organ dysfunction. Liver damage was defined by alanine or aspartate aminotransferase levels above the upper limit of normal. Myocardial injury was defined as a cardiac troponin concentration above the upper limit of normal. Altered mental status was defined as either delirium or delayed awakening after the discontinuation of sedation. Sepsis was defined as SIRS with a proven or suspected source of infection. Shock was defined as a systolic blood pressure ≤90 mmHg. Bowel obstruction was diagnosed on the basis of the following criteria: plain X-ray or abdominal CT indicating obstruction, patient presented with abdominal pain, vomiting, abdominal distension, and the absence of gas and stool for more than 24 h. The clinical outcomes included organ failure, length of ICU stay, length of hospital stay, and mortality.

### 2.3. Sample Collection, DNA Extraction, and 16S rRNA Gene Sequencing

The fecal samples were collected by sterile rectal swab on the first day of hospital admission. Details of sample collection were reported in previous studies [14,22]. The following steps were applied during sampling. Firstly, the head of a sterile dry swab was moistened with sterile saline. Secondly, the moistened swab was gently inserted approximately 4–5 cm into the anus and then rotated several times, to collect microorganisms from the rectal mucosal surface. Thirdly, the swab head was cut off and placed in a sterilized tube. Fourthly, the samples were stored at −80 °C refrigerator before analysis. Microbial genomic DNA was extracted from fecal samples using the bead-beating method that was based on a previously described protocol [23]. The extracted DNA was subjected to agarose gel electrophoresis to detect the concentration and purity. An appropriate amount of DNA sample was placed in a sterile centrifuge tube and then diluted to 1 ng/μL with sterile water. The V3–V4 region of the 16S rRNA was amplified by polymerase chain reaction (PCR) and then purified, as described in the previous study [24]. The diluted DNA served as a template, and primers with Barcode, Phusion^®^ High-Fidelity PCR Master Mix with GC Buffer (New England Biolabs, Ipswich, MA, USA), and efficient and high-fidelity enzymes were used for PCR. A sequencing library of V3–V4 regions of the 16S rRNA gene was prepared, as previously described [25] using the TruSeq^®^ DNA PCR-Free Sample Preparation Kit. The purified amplicons were pooled and sequenced on an Illumina MiSeq platform (Illumina Inc., San Diego, CA, USA).

### 2.4. Bioinformatics Analyses

The downstream amplicon bioinformatic analyses were performed based on the EasyAmplicon (Version 1.10) [26]. The software package VSEARCH (Version 2.7.1) [27] was used for dereplication. These unique sequences were denoised into amplicon sequence variants (ASV) with the unoise3 algorithm being implemented in USEARCH (Version 10.0) [28], and an ASV table was generated with the --usearch_global function. Taxonomic classification of ASVs was achieved using the sintax algorithm of USEARCH based on the Ribosomal Database Project (RDP) training set v16 [29].

### 2.5. Statistical Analysis

The microbial diversity was analyzed using R software (Version 4.0.3). Alpha diversity analyses included richness and Shannon index, which were accomplished by the vegan package (Version 2.5-7) [30]. Beta diversity was calculated by the Principal Coordinate Analysis (PCoA) based on weighted UniFrac distance of microbial communities. The Turkey Honestly significant difference (HSD) test and Adonis test were used to analyze significant differences between the groups for alpha and beta diversity, respectively. The results of diversity analyses and rarefaction curves were visualized by the ggplot2 package, and the Venn diagram illustrating ASV overlapping between groups was generated using the VennDiagram package. 

The compositions of the microbial community in two groups were presented as stacked bar plots at the phylum, family, and genus level, respectively. A chord diagram was used to perform the relationship between taxonomic level of class and different groups using the circlize package. The maptree showing the hierarchical relationship of classification was accomplished based on ggraph package (Version 2.0.4) [31]. A Manhattan plot and a volcano plot showing differential ASVs between groups at the phylum level through the R package, respectively. The edgeR package [32] was utilized to evaluate the differences in ASV abundance between HTGAP and non-HTGAP groups, and the Benjamini–Hochberg method was used to control the false discovery rate (FDR).

The Bugbase [33] and Phylogenetic Investigation of Communities by Reconstruction of Unobserved States (PICRUSt2) [34] were used to predict the phenotypes and functional pathways of the bacterial community, respectively. The Kruskal–Wallis test was used to evaluate the differential abundance of metabolic pathways between groups based on STAMP (Version 2.1.3) software, and Storey FDR [35] was used to correct for multiple comparisons. The correlations between variables, including hypertriglyceridemia, ASVs, metabolic pathways, disease severity, and clinical outcomes, were performed using the Spearman nonparametric correlation analysis and then visualized using the pheatmap package (Version 1.0.12) [36].

Analyses of the clinical characteristics in two groups were performed using SPSS (Version 25.0). The data were presented as mean ± standard deviation (SD) for continuous variables with normality, as median (Interquartile Range [IQR]) for abnormally distributed continuous variables, and as number (percentages) for categorical variables. The P values were calculated by the Chi-square or Fisher test for categorical variables. The t-test or non-parametric Kruskal–Wallis was used for continuous variables. A two-sided P value of less than 0.05 was considered to be statistically significant.

## 3. Results

### 3.1. Clinical Characteristics

A total of 60 patients were enrolled in this study and they were divided into two groups based on triglyceride level. Table 1 shows the comparison of demographic and clinical characteristics between the HTGAP and the non-HTGAP groups. Patients were younger (40.8 ± 11.3 vs. 51.8 ± 16.6; *p* = 0.004), had higher body mass index (BMI) (27.6 ± 3.7 vs. 24.7 ± 2.3; *p* = 0.001), and had a higher proportion of fatty liver (86.7% vs. 50.0%; *p* = 0.002) in the HTGAP group as compared with those in the non-HTGAP group. The HTGAP group also showed a higher CRP level (median 160.0, IQR 131.8–232.1 vs. median 106.9, IQR 21.5–160.0; *p* = 0.002).

Furthermore, patients in the HTGAP group had a higher risk of developing SAP (46.7% vs. 20.0%; *p* = 0.028), higher incidence of systemic inflammatory response syndrome (SIRS) (63.3% vs. 36.7%; *p* = 0.039), acute respiratory distress syndrome (ARDS) (50.0% vs. 23.3%; *p* = 0.032), organ failure (56.7% vs. 30.0%; *p* = 0.037), ICU admission (53.3% vs. 20.0%; *p* = 0.007), and had a longer hospital stay (median 18.0, IQR 9.8–28.0 vs. median 6.5, IQR 3.0–14.0; *p* = 0.001) as compared with patients in non-HTGAP group.

### 3.2. Complications of HTGAP Patients Affects Gut Microbiome Composition

Sixty rectal swabs were collected and sent for sequencing. No sample was discarded due to poor sequencing quality. A total of 1,565,127 reads were detected from samples and assigned to 22,979 ASVs. On average, each sample has 383 ASVs (range 68–583) and 26,085 sequences (range 5333–46,528) (Table S1). The Venn diagram analysis revealed that there were 277 and 213 ASVs observed in non-HTGAP and HTGAP groups, respectively, and these two groups shared 40 ASVs in common (Figure S1).

**Figure 1:**
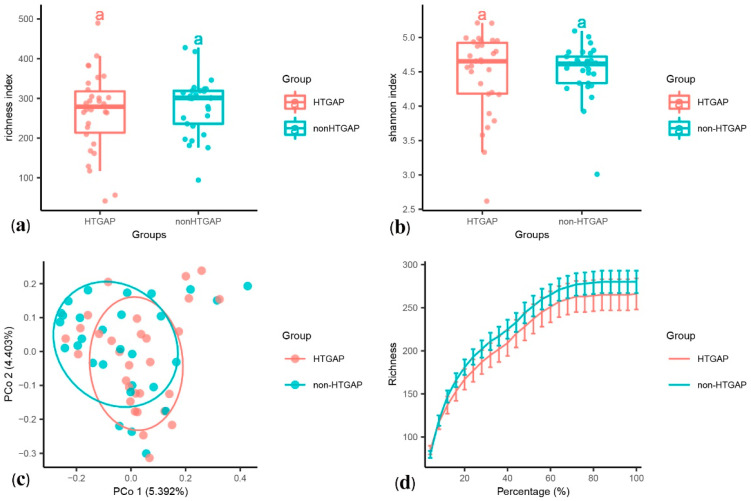
Alpha and beta diversity analysis between HTGAP and non-HTGAP groups. (**a**) Alpha diversity based on species richness. (**b**) Alpha diversity based on Shannon’s diversity index. Box-plot features represent the median (central line), upper and lower quartiles (box), and the maximum and minimum values of the data (bars). (**c**) Beta diversity analysis based on PCoA plot. The abscissa and ordinate represent the contribution rate of the first principal component (5.392%) and the second principal component (4.403%) to the sample difference. Each symbol represents the gut microbiota of a sample. (**d**) Rarefaction curve of species richness. HTGAP, hypertriglyceridemia-associated acute pancreatitis; PCoA, principal coordinate analysis.

The alpha diversity was assessed using species richness (Figure 1a) and Shannon’s diversity index (Figure 1b). We found no statistically significant difference in the species richness (*p* = 0.440) and Shannon’s diversity index (*p* = 0.571) between the HTGAP and non-HTGAP groups, but there was a tendency for reduced richness in the HTGAP group. The PCoA of weighted UniFrac distances for the beta-diversity assessment did not show significant differences in the microbial communities between the HTGAP and non-HTGAP groups (Figure 1c). The rarefaction curves (Figure 1d) of species richness gradually flattened out, which indicated that a reasonable number of individual samples had been taken. 

**Figure 2:**
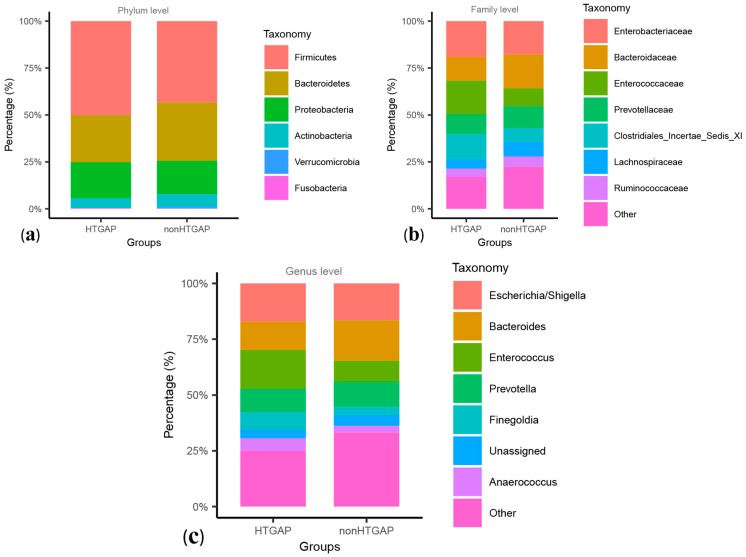
Relative abundances of bacteria between groups at the phylum (**a**), family (**b**), and genus (**c**) levels. Bacteria that below 1.00% in two groups or cannot be assigned to a specific taxonomic category, were grouped into others. HTGAP, hypertriglyceridemia-associated acute pancreatitis.

These two groups were composed mainly of Firmicutes, Bacteroidetes, and Proteobacteria at the level of phylum, and Firmicutes were more frequent in the HTGAP group than in the non-HTGAP group, as shown in Figure 2a. At the family level, *Enterococcaceae* and *Clostridiales Incertae Sedis XI* were more abundant in the HTGAP group, while *Lachnospiraceae* and *Bacteroidaceae* were more abundant in the non-HTGAP group (Figure 2b). At the genus level, the HTGAP group showed a higher relative abundance of *Finegoldia* and *Enterococcus*, and a lower relative abundance of *Bacteroides* when compared with the non-HTGAP group (Figure 2c). A visualization of connections between microbiota composition and groups of patients is depicted in the chord diagram (Figure S2), demonstrating a higher abundance of *Bacilli* in the HTGAP group as compared to the non-HTGAP group. Figure S3 shows the level structure of intestinal microflora.

### 3.3. Taxonomic Features Are Different in Patients with Non-HTGAP and HTGAP

**Figure 3:**
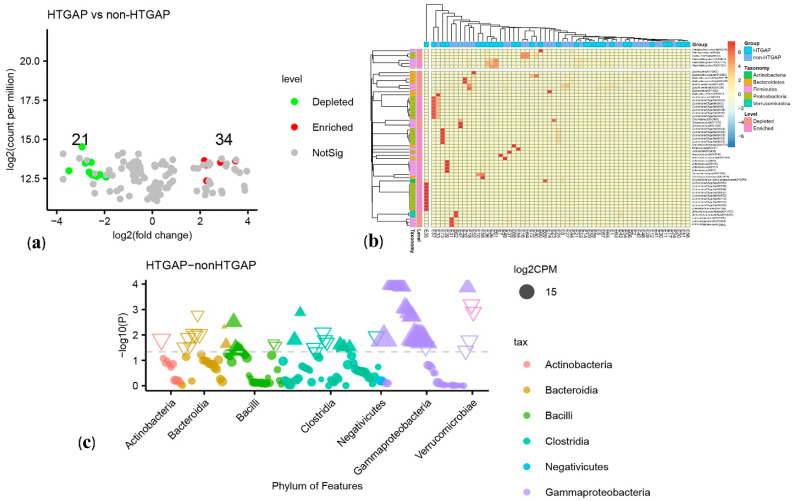
Differential ASVs between HTGAP and non-HTGAP groups. ASV, amplicon sequence variants. (**a**) Volcano plot. Each point represents an ASV, and significantly different ASVs are colored (green = depleted in the non-HTGAP group; red = enriched in the non-HTGAP group; gray = not significantly enriched or depleted). (**b**) Heatmap. Rows represent discriminative ASVs, and columns represent individual samples. Blue represents lower relative abundances, and red represents higher relative abundances. The taxonomic assignment and level of abundance (enriched or depleted) are provided to the left of the heatmap. (**c**) Manhattan plot. The x axis represents the microbial ASV taxonomy at class level ranked by alphabetical order, and y axis represents -log10 (p value). Filled triangles, hollow inverted triangles, and solid circles indicate ASVs enriched, depleted, and without significant difference, respectively. The color of each marker represents the different taxonomic affiliation of the ASVs, and the size corresponds to their relative abundances using log2 transformed CPM values. HTGAP, hypertriglyceridemia-associated acute pancreatitis; ASV, amplicon sequence variants; CPM, count per million.

**Figure 4:**
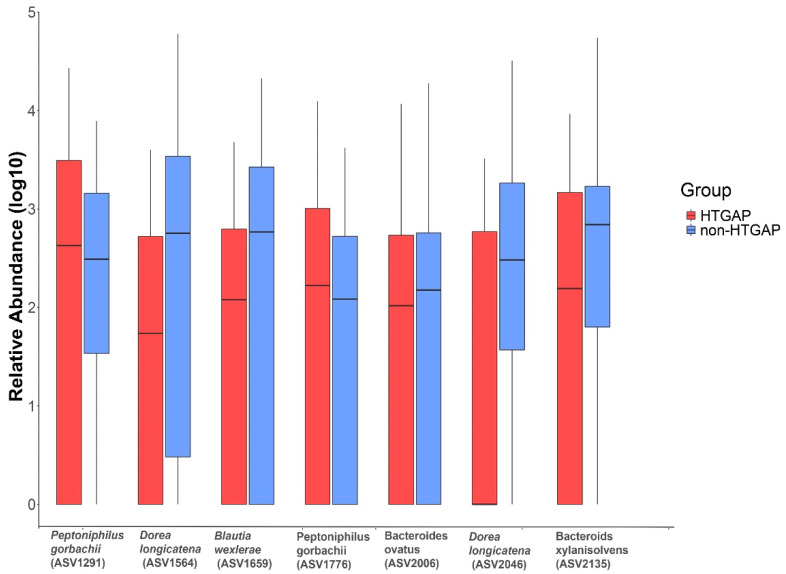
Relative abundances of differential ASVs between HTGAP and non-HTGAP groups. Box-plot features represent the median (central line), upper and lower quartiles (box), and the maximum and minimum values of the data (bars). HTGAP, hypertriglyceridemia-associated acute pancreatitis; ASV, amplicon sequence variants.

The volcano plot (Figure 3a) and heatmap (Figure 3b) depicted 55 ASVs with significantly different read counts between HTGAP and non-HTGAP groups based on the edgeR analysis, among which 21 ASVs were depleted and 34 ASVs were enriched in HTGAP group as compared with non-HTGAP group. At the phylum level, *Actinobacteria* and *Bacteroidia* were enriched, while *Bacilli*, *Clostridia*, and *Gammaproteobacteria* were depleted in the non-HTGAP group compared to HTGAP group (Figure 3c). At the species level, HTGAP group had higher abundances of *Peptoniphilus gorbachii* (ASV1291) (0.333% vs. 0.126%, *p* = 0.031) and *Peptoniphilus gorbachii* (ASV1776) (0.153% vs. 0.057%, *p* = 0.029), but lower abundances of *Dorea longicatena* (ASV1564) (0.066% vs. 0.437%, *p* = 0.008), *Dorea longicatena* (ASV2046) (0.044% vs. 0.240%, *p* = 0.020), *Blautia wexlerae* (ASV1659) (0.064 vs. 0.218, *p* = 0.046), *Bacteroides ovatus* (ASV2006) (0.073% vs. 0.131%, *p* = 0.030), and *Bacteroides xylanisolvens* (ASV2135) (0.112 vs. 0.360, *p* = 0.009) when compared with non-HTGAP group (Figure 4).

### 3.4. Microbial Functions Altered in HTGAP Patients

**Figure 5:**
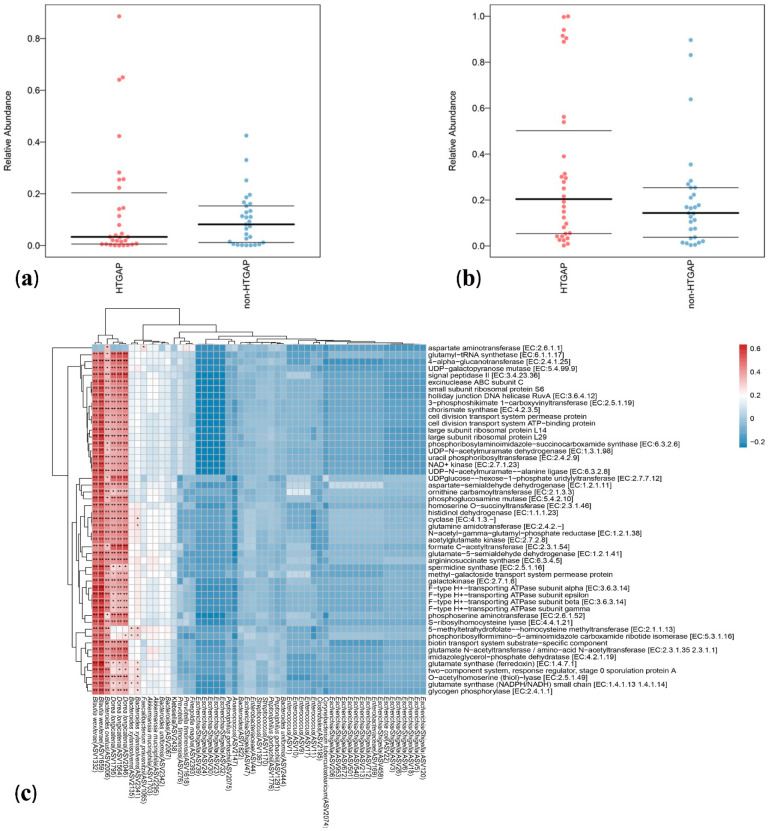
Predicted phenotypes and functional pathways of intestinal microflora. (**a**) Relative abundance of bacteria tolerant of oxidative stress. (**b**) Relative abundance of bacteria containing mobile elements. (**c**) Spearman correlations between HTGAP associated ASVs and functional pathways. Blue represents a negative correlation, and red represents a positive correlation. HTGAP, hypertriglyceridemia-associated acute pancreatitis; ASV, amplicon sequence variants. The strength of positive (red) or negative (blue) correlation is shown by two-color heatmap, with asterisks denoting statistical significance (* *p* < 0.05, ** *p* < 0.01).

Bugbase analysis was used to explore the differences in bacterial metabolic phenotypes between HTGAP and non-HTGAP groups, and the predictions of oxidative stress tolerance and mobile element containing are shown in Figure 5a,b, respectively. In contrast to the non-HTGAP group, the relative abundance of bacteria containing mobile elements slightly increased in the HTGAP group, but without significance. For oxidative stress, a lower abundance of bacteria with the ability to tolerate stress was detected in the HTGAP group. To analyze the metabolic functional pathways of intestinal microflora, PICRUSt2 was used and the result is shown in Figure 5c. *Blautia wexlerae* (ASV1332 and ASV1659), *Bacteroides ovatus* (ASV2006), and *Dorea longicatena* (ASV1795, ASV1564, and ASV2046) were positively correlated with microbial gene functions related to amino acid metabolism, biosynthesis of secondary metabolites, nucleotide biosynthesis, NAD+ kinase, glutamate synthase (NADPH/NADH) small chain, 4-alpha-glucanotransferase, formate C-acetyltransferase, and glycogen phosphorylase.

### 3.5. Associations between Clinical Indicators and Gut Microbiota

**Figure 6:**
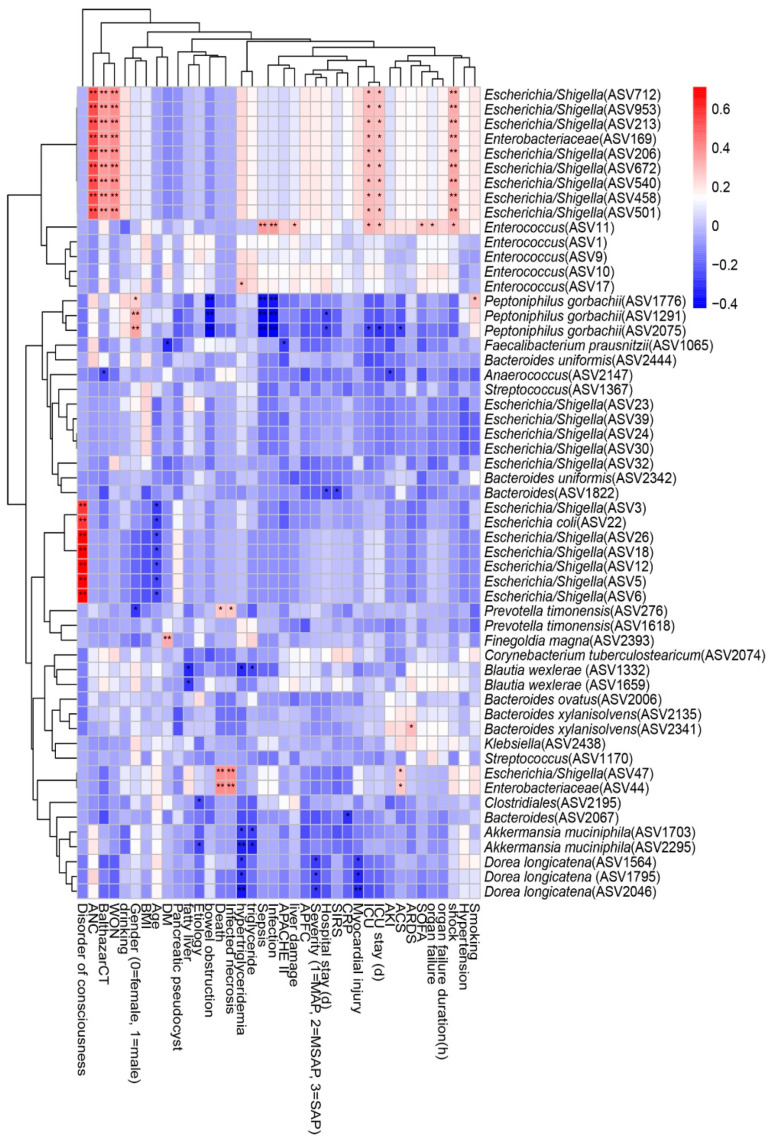
Spearman correlations between differential ASVs and clinical outcomes as well as indicators of disease severity. ASV, amplicon sequence variants; ANC, acute necrotic accumulation; WON, walled-off necrosis; BMI: body mass index; DM, diabetes mellitus; APACHE II: the Acute Physiology and Chronic Health Evaluation II score; APFC, acute peripancreatic fluid collection; MAP: mild acute pancreatitis; MSAP: moderately severe acute pancreatitis; SAP: severe acute pancreatitis; SIRS: systemic inflammatory response syndrome; CRP: C-reactive protein; ICU: intensive care unit; AKI: acute kidney injury; ACS: abdominal compartment syndrome; ARDS: acute respiratory distress syndrome; SOFA: the Sequential Organ Failure Assessment score. The strength of positive (red) or negative (blue) correlation is shown by two-color heatmap, with asterisks denoting statistical significance (* *p* < 0.05, ** *p* < 0.01).

We performed a Spearman correlation analysis to explore the relationship between intestinal microflora and clinical indicators such as disease severity and clinical outcomes, and the result is shown in Figure 6. We found that nine ASVs within *Escherichia/Shigella*, which belong to the family *Enterobacteriaceae*, had moderate to strong positive correlations with shock, ICU admission, ICU stay, acute necrotic accumulation, Balthazar score, and walled-off necrosis. *Enterococcus* within *Enterococcaceae* was positively correlated with sepsis, infection, liver damage, ICU admission, ICU stay, SOFA score, organ failure, and shock, but *Peptoniphilus gorbachii* within *Peptoniphilaceae* presented a negative correlation. Infected necrosis, a disorder of consciousness, and death were strongly positively correlated with *Escherichia/Shigella* within *Enterobacteriaceae*. Hypertriglyceridemia, myocardial injury, and disease severity were negatively correlated with *Akkermansia muciniphila* within *Verrucomicrobiaceae* and *Dorea longicatena* within *Lachnospiraceae*.

## 4. Discussion

In this study, we investigated the relationship between gut microbiota and the severity and prognosis of patients with HTGAP. We found that patients in the HTGAP group had a worse outcome than that those in the non-HTGAP group, and significant correlations existed between the altered intestinal microflora and prognostic markers, including disease severity, local and systemic complications, ICU admission, and mortality.

To our knowledge, this is the first study to investigate the relationship between intestinal flora and prognosis among patients with HTGAP. Consistent with previous studies [17,37], patients were younger in the HTGAP group when compared with those in the non-HTGAP group. The specific mechanisms by which hyperglycemia exacerbates AP remain unclear, and the most widely accepted explanation is that excessive production of free fatty acids leads to oxidative stress, vascular endothelial injury, pancreatic necrosis, and systemic inflammatory response [10,38]. Besides, HTGAP patients were more likely to be accompanied with fatty liver when compared with non-HTGAP patients, and previous studies found that fatty liver probably aggravated AP through the peroxisome proliferator activated receptor alpha (PPARα) signaling pathway and fatty acid degradation pathway [39,40].

We performed bioinformatics analysis to examine microbial diversity, richness, and community structure. Consistent with a previous study [41], *Firmicutes*, *Bacteroidetes*, and *Proteobacteria* were the most abundant phyla in the AP patients. In the present study, the microbial diversity in the HTGAP group, especially species richness, was lower than that in the non-HTGAP group, and lower microflora richness was related to adiposity, insulin resistance, dyslipidemia, and a more inflammatory state [42]. We postulate that the decrease in alpha diversity may lead to intestinal dysbiosis and disrupt barrier function, which contributes to a poor prognosis for AP patients. 

At the family level, *Lachnospiraceae* and *Bacteroidaceae* were less abundant in the HTGAP group when compared to the non-HTGAP group. Some *Lachnospiraceae* species, including *Dorea longicatena* and *Blautia wexlerae*, can ferment carbohydrates to produce SCFAs, which are mainly composed of acetate, propionate, and butyrate [43,44]. SCFAs are important sources of energy for intestinal mucosa and they regulate energy metabolism, inflammatory reaction, and intestinal homeostasis by different signaling pathways [45]. It was previously shown that *Blautia wexlerae* could contribute to the maintenance of intestinal immune homeostasis, and its depletion was associated with obesity and metabolic complications [46]. The study conducted by Mondot et al. found that the increase of *Dorea longicatena* was beneficial to maintaining the remission of Crohn’s disease [47]. These results suggested that the decrease in gut microflora which produced SCFAs may be responsible for the poor outcome of HTGAP patients. Some *Bacteroides* species within the family *Bacteroidaceae*, including *Bacteroides ovatus*, *Bacteroides xylanisolvens*, and *Bacteroides uniformis*, were essential species to induce the production of gut IgA, which acted as a protective factor for maintaining the stability of the intestinal environment by limiting the bacteria and endotoxin invasive intestinal epithelial cells, facilitating bacterial clearance, and regulating bacterial colonization [48,49,50].

At the genus level, *Finegoldia* and *Enterococcus* had a higher relative abundance in the HTGAP group when compared with the non-HTGAP group. *Escherichia/Shigella* and *Enterococcus* were the most common bacterial pathogens for infected pancreatic necrosis, which were significantly associated with the poor prognosis of AP patients [21]. Consistent with previous studies, an increase in the relative abundance of *Enterococcus* was related to higher disease severity [12,14]. In addition, we found that *Peptoniphilus* and *Anaerococcus* were more abundant in the HTGAP group, while *Akkermansia* was more abundant in the non-HTGAP group. *Anaerococcus*, *Peptoniphilus gorbachii*, and *Finegoldia magna*, like many opportunistic pathogenic bacteria, were able to cause bacterial translocation and infected necrosis that may aggravate AP. *Akkermansia muciniphila* was important not only for the regulation of mucus layer thickness and gut barrier integrity, but also for improving the immune system and lipid metabolism [51,52].

Besides, there were significant differences in gut microbial metabolic phenotypes between the HTGAP and non-HTGAP groups. The relative abundance of bacteria containing mobile elements in HTGAP was significantly higher than that in non-HTGAP, which might be a marker for translocation of gut microflora. Besides, stress-tolerant bacteria were less abundant in the HTGAP group as compared to the non-HTGAP group, indicating that intestinal flora in the HTGAP group was more difficult to tolerate oxidative stress and maintain mucosal barrier function. We analyzed the signaling pathways using the Kyoto Encyclopedia of Genes and Genomes (KEGG) database to characterize the functional role of the intestinal flora. We found that *Blautia wexlerae*, *Bacteroides ovatus*, and *Dorea longicatena* were positively correlated with microbial gene functions that were related to amino acid metabolism, biosynthesis of secondary metabolites, nucleotide biosynthesis, NAD+ kinase, carbohydrate, and energy metabolism, and formate C-acetyltransferase. Amino acid, carbohydrate, and energy metabolism were the primary life activities of bacteria, which provided a basis for the stress tolerance mechanism. Nucleotide coenzymes, including NAD+, NADP+, NADH, and NADPH, were closely associated with energy metabolism, antioxidation stress, and immunological functions [53]. Formate C-acetyltransferase converted pyruvate to formate and acetyl coenzyme A, which were subsequently metabolized to butyrate. This analysis suggested that *Blautia wexlerae*, *Bacteroides ovatus*, and *Dorea longicatena* could be potential probiotics, as they could defend against oxidative stress, regulate the production of SCFAs, and maintain gut homeostasis.

There are several limitations to this study. Firstly, we investigated the association between the prognosis of HTGAP patients and intestinal microflora at a single time point, lacking longitudinal monitoring of the changes in intestinal microflora with disease development. Secondly, the sample size was relatively small, and it was from a single center. Multicenter studies with a larger sample size are required to test our findings. Thirdly, our study was based on 16S rRNA gene sequencing rather than the entire genomes, which provides limited information regarding bacterial genes and their functions.

Our study revealed that patients with HTGAP had altered gut microbiome, which, in turn, was associated with disease exacerbation and poor prognosis. The correlation analysis and functional prediction analysis provided insights into disease progression in AP patients complicated with hypertriglyceridemia. We speculate that the HTGAP caused gut microecological disturbance, which may take an important role in the worsening of acute pancreatitis. Further validation and exploration via functional studies are required to prove the causal relationship.

## 5. Conclusions

In conclusion, there were significant differences in the composition and function of gut microbiota in HTGAP patients compared to non-HTGAP patients, and changes of intestinal microflora were related to poor prognosis of HTGAP. Further studies are needed to determine the mechanisms underlying the effects of the microbiome on mucosal barrier and inflammatory response in HTGAP patients.

## Figures and Tables

**Table 1:** Demographic and clinical characteristics of two groups.

Variables	HTGAP (n = 30)	Non-HTGAP (n = 30)	*p*
Age (years), mean (SD)	40.8 (11.3)	51.8 (16.6)	0.004
Male, n (%)	17 (56.7)	14 (46.7)	0.438
BMI (kg/m^2^), mean (SD)	27.6 (3.7)	24.7 (2.3)	0.001
Overweight (BMI 25–29.9 kg/m^2^), n (%)	19 (63.3)	13 (43.3)	0.121
Obesity (BMI ≥ 30 kg/m^2^), n (%)	5 (13.3)	0 (0.0)	0.020
Smoking, n (%)	11 (36.7)	6 (20.0)	0.152
Drinking, n (%)	10 (33.3)	5 (16.7)	0.136
Comorbid abnormalities, n (%)
Hypertension	10 (33.3)	11 (36.7)	0.787
Diabetes	8 (26.7)	6 (20.0)	0.542
Fatty liver	26 (86.7)	15 (50.0)	0.002
Laboratory examinations
Triglyceride (mmol/L), median (IQR)	18.7 (14.0, 34.9)	1.2 (0.6, 1.9)	<0.001
CRP (mg/L), median (IQR)	160.0 (131.8, 232.1)	106.9 (21.5, 160.0)	0.002
Etiology, n (%)
Biliary	0 (0.0)	25 (83.3)	<0.001
Hypertriglyceridemia	30 (100.0)	0 (0.0)	<0.001
Alcohol consumption	0 (0.0)	5 (16.7)	0.026
APACHE Ⅱ, median (IQR)	6.0 (3.0, 11.0)	5.0 (2.0, 7.25)	0.147
SOFA score, median (IQR)	2.0 (0.0, 4.3)	1.0 (0.0, 3.25)	0.187
Balthazar score E, n (%)	6 (20.0)	3 (10.0)	0.278
Disease severity, n (%)
MAP	6 (20.0)	14 (46.7)	0.028
MSAP	10 (33.3)	10 (33.3)	1.000
SAP	14 (46.7)	6 (20.0)	0.028
Local complications, n (%)
APFC	21 (70.0)	14 (46.7)	0.067
Pancreatic pseudocyst	5 (16.7)	4 (13.3)	0.718
ANC	3 (10.0)	1 (3.3)	0.301
WON	2 (6.7)	0 (0.0)	0.150
Infected necrosis	1 (3.3)	0 (0.0)	0.313
Systematic complication, n (%)
SIRS	19 (63.3)	11 (36.7)	0.039
ARDS	15 (50.0)	7 (23.3)	0.032
AKI	8 (26.7)	5 (16.7)	0.347
Shock	4 (13.3)	5 (16.7)	0.718
ACS	5 (16.7)	2 (6.7)	0.228
Liver damage	5 (16.7)	6 (20.0)	0.739
Myocardial injury	4 (13.3)	1 (3.3)	0.161
Altered mental status	1 (3.3)	0 (0.0)	0.313
Sepsis	7 (23.3)	6 (20.0)	0.754
Bowel obstruction	8 (26.7)	3 (10.0)	0.095
Outcome
Organ failure, n (%)	17 (56.7)	9 (30.0)	0.037
Organ failure duration (h), median (IQR)	72.0 (48.0, 276.0)	50.0 (33.5, 121.0)	0.465
ICU, n (%)	16 (53.3)	6 (20.0)	0.007
ICU stay (days), median (IQR)	9.0 (5.3, 14.5)	6.5 (5.8, 8.8)	0.395
Hospital stay (days), median (IQR)	18.0 (9.8, 28.0)	6.5 (3.0, 14.0)	0.001
Death, n (%)	1 (3.3)	0 (0.0)	0.313

HTGAP, hypertriglyceridemia-associated acute pancreatitis; SD, standard deviation; BMI: body mass index; CRP: C-reactive protein; IQR, interquartile range; APACHE II: the Acute Physiology and Chronic Health Evaluation II score; SOFA: the Sequential Organ Failure Assessment score; MAP: mild acute pancreatitis; MSAP: moderately severe acute pancreatitis; SAP: severe acute pancreatitis; APFC, acute peripancreatic fluid collection; ANC, acute necrotic accumulation; WON, walled-off necrosis; ARDS: acute respiratory distress syndrome; AKI: acute kidney injury; ACS: abdominal compartment syndrome; SIRS: systemic inflammatory response syndrome; ICU: intensive care unit.

## Data Availability

The data presented in this study are available on request from the corresponding author. The data are not publicly available due to the small possibility of compromising the individual privacy of patients.

## Supplementary Materials

The following are available online at https://www.mdpi.com/article/10.3390/biom11050695/s1, Table S1: ASV table summary, Figure S1: a Venn diagram demonstrating the existence of ASVs with a relative abundance > 0.1% in two groups, Figure S2: a chord diagram showing connections between microbiota composition and groups of patients, Figure S3: a maptree showing the phylogenetic relationship among intestinal microflora at phylum level.
