# Supplementary material for: Variations in Gut Microbiome Are Associated with Prognosis of Hypertriglyceridemia-Associated Acute Pancreatitis
Source: Biomolecules. 2021 May 6;11(5):695. doi: 10.3390/biom11050695 (PMC8148198; doi:10.3390/biom11050695)
Supplement: Supplementary file 1 [file biomolecules-11-00695-s001.zip › biomolecules-1199177-supplementary.pdf]

Variations in gut microbiome are associated with prognosis of hypertriglyceridemia-associated acute pancreatitis

Short Title: Gut microbiome and prognosis of HTGAP

Xiaomin Hu[1#], Liang Gong[2#], Ruilin Zhou[3#], Ziying Han[2], Li Ji[2], Yan Zhang[4], Shuyang Zhang[3], Dong Wu[2*]

[1] Department of Gastroenterology, Department of Medical Research Center, State Key Laboratory of Complex Severe and Rare Diseases, Peking Union Medical College Hospital, Chinese Academy of Medical Science & Peking Union Medical College, 100730, Beijing, China.

[2] Department of Gastroenterology, State Key Laboratory of Complex Severe and Rare Diseases, Peking Union Medical College Hospital, Chinese Academy of Medical Sciences & Peking Union Medical College, 100730, Beijing, China

[3] Department of Cardiology, Peking Union Medical College Hospital, Chinese Academy of Medical Science & Peking Union Medical College, 100730, Beijing, China.

[4] Institute of Cardiovascular Sciences and Key Laboratory of Molecular Cardiovascular Sciences, School of Basic Medical Sciences, Ministry of Education, Peking University Health Science Center, Beijing, China.

[#] These authors contributed equally to this work.

[*] Correspondence to Dong Wu, MD. Department of Gastroenterology, State Key Laboratory of Complex Severe and Rare Diseases, Peking Union Medical College Hospital, Chinese Academy of Medical Sciences & Peking Union Medical College, No.1 Shuaifuyuan, 100730, Beijing, China. E-Mail: wudong@pumch.cn

Supplementary Table

Table S1. ASV table summary

Sample ID	Group	Sequence counts	ASV number
E1	HTGAP	32402	406
E10	HTGAP	8671	553
E11	HTGAP	12549	271
E12	HTGAP	14150	257
E14	HTGAP	41907	396
E16	Non-HTGAP	38287	446
E17	HTGAP	15237	179
E18	Non-HTGAP	9243	351
E2	Non-HTGAP	31212	459
E20	HTGAP	38993	569
E21	HTGAP	41410	551
E23	Non-HTGAP	10974	372
E24	Non-HTGAP	10771	236
E25	HTGAP	33704	407
E26	HTGAP	38360	483
E27	Non-HTGAP	33508	378
E28	Non-HTGAP	33664	370
E29	HTGAP	10992	427
E30	Non-HTGAP	32272	409
E31	Non-HTGAP	32962	300
E32	Non-HTGAP	36101	484
E33	Non-HTGAP	31336	583
E35	HTGAP	12785	197
E36	HTGAP	13178	403
E37	Non-HTGAP	28800	408
E39	HTGAP	39409	68
E40	Non-HTGAP	35831	337
E41	Non-HTGAP	15577	134
E42	Non-HTGAP	8962	327
E43	Non-HTGAP	30759	408
E44	Non-HTGAP	30039	425
E47	Non-HTGAP	6682	332
E48	Non-HTGAP	12798	406
E49	Non-HTGAP	21060	582
E5	HTGAP	7746	318
E51	HTGAP	40852	480
E52	Non-HTGAP	34009	398
E53	Non-HTGAP	12435	350
E54	HTGAP	31095	394
E55	HTGAP	43515	276
E56	HTGAP	37401	476

E57	HTGAP	41914	74
E58	HTGAP	14265	326
E59	HTGAP	12131	505
E6	Non-HTGAP	35773	566
E60	HTGAP	14933	430
E61	HTGAP	40959	425
E62	Non-HTGAP	20419	439
E63	Non-HTGAP	31289	318
E64	Non-HTGAP	35677	299
E65	Non-HTGAP	9250	355
E66	HTGAP	14800	404
E67	HTGAP	25179	542
E68	HTGAP	46528	383
E69	Non-HTGAP	34924	370
E7	HTGAP	40944	448
E72	HTGAP	5333	227
E73	HTGAP	30122	314
E74	Non-HTGAP	35340	493
E8	Non-HTGAP	23709	455

ASV, amplicon sequence variants; HTGAP, hypertriglyceridemia-associated acute pancreatitis.

Supplementary Figures

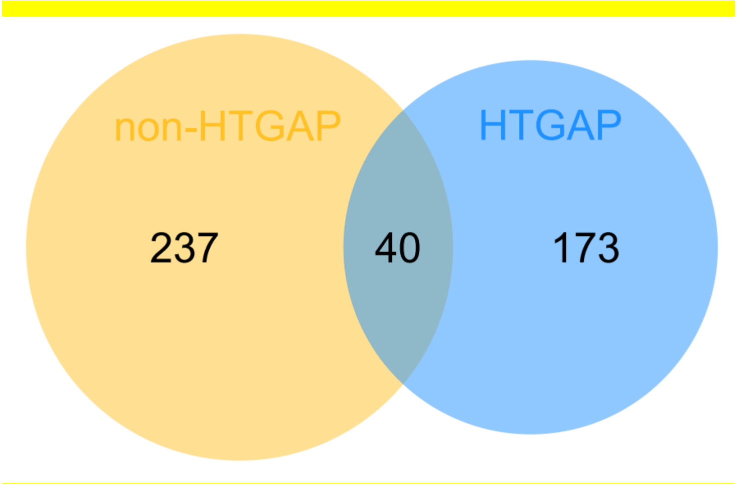

Figure S1. A Venn diagram demonstrating the existence of ASVs with a relative abundance > 0.1% in two groups. ASV, amplicon sequence variants.

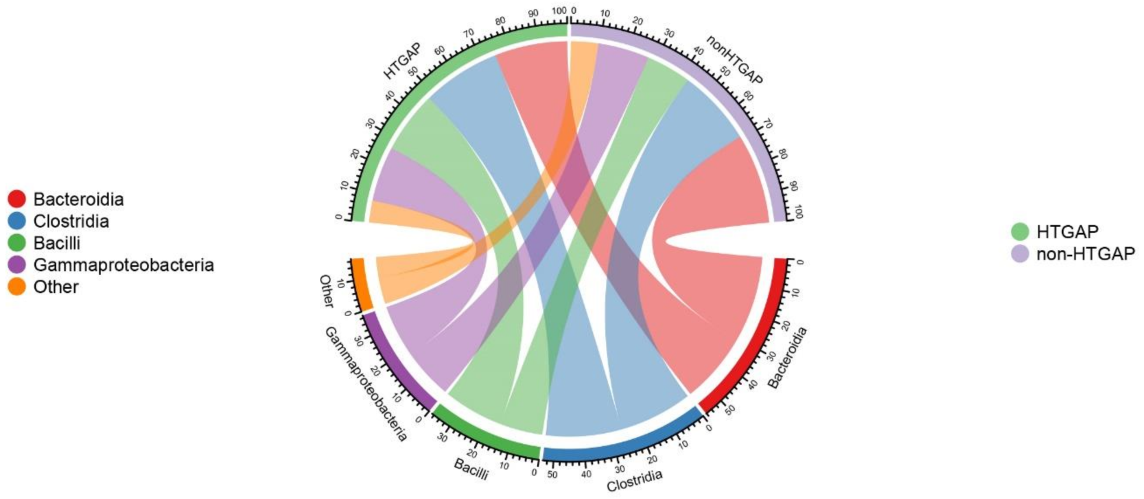

Figure S2. A chord diagram showing connections between microbiota composition and groups of patients. The width of the chords is determined by the relative abundance of bacteria.at class level. HTGAP, hypertriglyceridemia-associated acute pancreatitis.

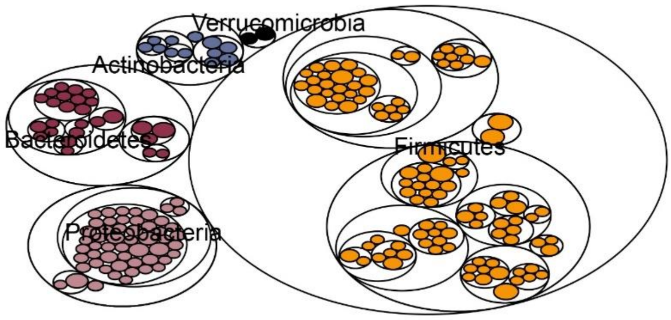

Figure S3. A maptree showing the phylogenetic relationship among intestinal microflora at phylum level. The largest circle represented the phylum level, and the gradually shrinking circle represented the classification level from the family to the species. The diameter of each circle is proportional to the relative abundance of the taxon.
